# Rare Case of Internal Jugular Vein Thrombosis Associated With Squamous Cell Carcinoma of the Posterior Tongue

**DOI:** 10.7759/cureus.101757

**Published:** 2026-01-17

**Authors:** Ibikiri Okoye, Awais Altaf Shah, Urooj Zahra

**Affiliations:** 1 Hospital Medicine, United Lincolnshire Hospitals NHS Trust, Lincoln, GBR; 2 General Medicine, United Lincolnshire Hospitals NHS Trust, Lincoln, GBR; 3 Internal Medicine, Fatima Jinnah Medical University, Lahore, PAK

**Keywords:** cancer associated thrombosis, cat, ijvt, internal jugular vein thrombosis, provoked ijvt, provoked internal jugular vein thrombosis, scc of tongue, squamous cell carcinoma of tongue, venous thrombo-embolism, vte

## Abstract

Internal jugular vein thrombosis (IJVT) is an uncommon but potentially life-threatening condition, arising from diverse risk factors including infection, instrumentation, and malignancy. Although its incidence in head and neck (H&N) cancers remains low, early recognition is essential, as serious complications such as pulmonary embolism, stroke, and superior vena cava obstruction can arise.

We report the case of a 77-year-old man with a remote history of treated H&N malignancy, who had a new diagnosis of base-of-tongue squamous cell carcinoma (scc) after he developed a right-sided neck swelling. He showed initial improvement with palliative radiotherapy. He presented again a couple of months later with bilateral facial swelling extending to the right neck, initially treated as facial cellulitis. However, antibiotics proved ineffective, leading us to investigate further. Subsequent contrast-enhanced arterial-phase CT findings were in line with a bland right IJVT, for which anticoagulation was commenced promptly.

This case highlights the importance of considering provoked IJVT in patients with H&N SCC who present with persistent neck or facial swelling, even in the absence of other systemic risk factors. Early diagnostic imaging and timely anticoagulation may prevent potentially fatal thromboembolic complications.

## Introduction

Internal jugular vein thrombosis (IJVT) is a rare and serious condition, typically caused by central venous catheterisation, malignancy, and intravenous drug use [1]. It commonly presents as neck and arm oedema with associated pain. Although IJVT can occur on either side, most cases are reported on the left; our patient presented with right-sided thrombosis, which is less common [1]. Potentially life-threatening complications include pulmonary embolism, cerebral infarction, and superior vena cava obstruction [2]. We present a case of right internal jugular vein thrombosis associated with squamous cell carcinoma (SCC) of the posterior tongue, an underappreciated presentation in head and neck (H&N) oncology, which mimicked cellulitis in our patient and hence led to treatment delay.

## Case presentation

In November 2024, a 77-year-old man presented with a two-week history of bilateral lower facial oedema, right neck swelling and erythema. Vitals at presentation included temperature 36.8, blood pressure 130/91, oxygen saturation 94%, respiratory rate 22, heart rate 89 bpm, and regular pulse. On examination, there was obvious right lower facial oedema without upper limb oedema. Oral examination showed no notable findings. There was no significant lymphadenopathy, discrete measurable mass, or collection. The oedema was non-pitting, with no warmth, tenderness, fluctuance, lymphatic streaking, or skin breaks. The patient had a permanent tracheostomy tube in situ, which was clear of secretions and patent, and the stoma was clean. There was no venous distension, plethora, or collateral veins. There was no history of dysphagia or odynophagia.

**Figure 1:**
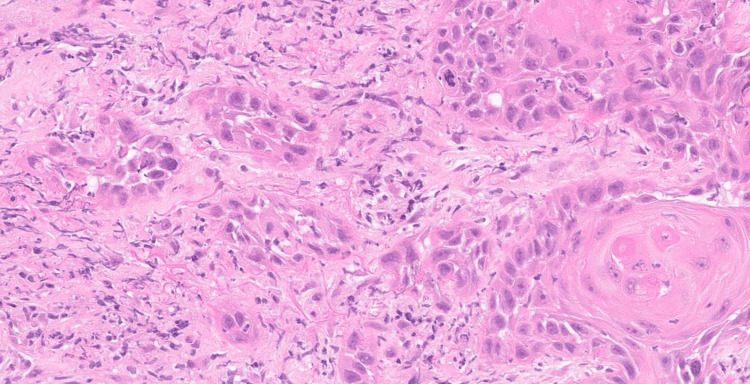
Histology slide showing multiple fragments of tissue infiltrated by moderately differentiated SCC Haematoxylin and eosin (H&E) stained tissue from the right base of the tongue (at x20 magnification). SCC: Squamous cell carcinoma

In 2007, the patient had a radical neck dissection with laryngectomy and a permanent tracheostomy for a H&N malignancy. The histology at the time showed a poorly differentiated carcinoma, with the differential of nasopharyngeal carcinoma and a SCC of the tongue with a basaloid morphology. Imaging had ruled out a nasopharyngeal carcinoma. A subsequent pan-endoscopic biopsy of the base of the tongue was unremarkable. In early 2024, he had an MRI for new onset swelling of the right side of his neck. It showed a right basal tongue lesion extending to the pharyngeal tonsillar region. The PET scan showed abnormal uptake in the same region. An endoscopic biopsy was taken from the right base of the tongue, and we had our first true diagnosis of a right base of tongue SCC on biopsy (Figure 1).

Although it could represent a recurrence or a new primary malignancy, given the long interval between the two presentations and different subsites of the tongue each time, the new lesion seemed to be consistent with a new primary. The human papillomavirus (HPV)/p16 testing was not performed, as it would not have altered planned palliative management. Due to a decline in his performance status, it was deemed that radiotherapy would be in his best interest. The patient tolerated that well, and his signs and symptoms improved. However, he presented about two months later with recurrent facial swelling, bilateral this time, extending to the right side of his neck.

Baseline laboratory investigations are summarised in Table 1. These demonstrated a markedly raised C-reactive protein (CRP) with a normal white blood cell count, normal renal function, and a negative D-dimer as per age-adjusted reference values (which does not reliably exclude IJVT in the setting of malignancy). Sputum and blood cultures were negative.

**Table 1:** Laboratory investigations on admission CRP: C-reactive protein; eGFR: Estimated glomerular filtration rate; INR: International normalised ratio

Parameter	Result	Reference range
Haemoglobin	122 g/L	132–170 g/L
White blood cell count	5.4 × 10⁹/L	4.3–11.3 × 10⁹/L
Neutrophils	3.73 × 10⁹/L	2.1–7.4 × 10⁹/L
Lymphocytes	1.0 × 10⁹/L	1.0–3.6 × 10⁹/L
Platelets	275 × 10⁹/L	150–400 × 10⁹/L
CRP	103 mg/L	0–5 mg/L
Estimated glomerular filtration rate (eGFR)	>90 mL/min/1.73 m²	≥90 mL/min/1.73 m²
International normalised ratio (INR)	1.0	0.9–1.1
D-dimer	221 ng/mL	<230 ng/mL (age-adjusted)
Blood cultures	Negative	—
Sputum cultures	Negative	—

**Figure 2:**
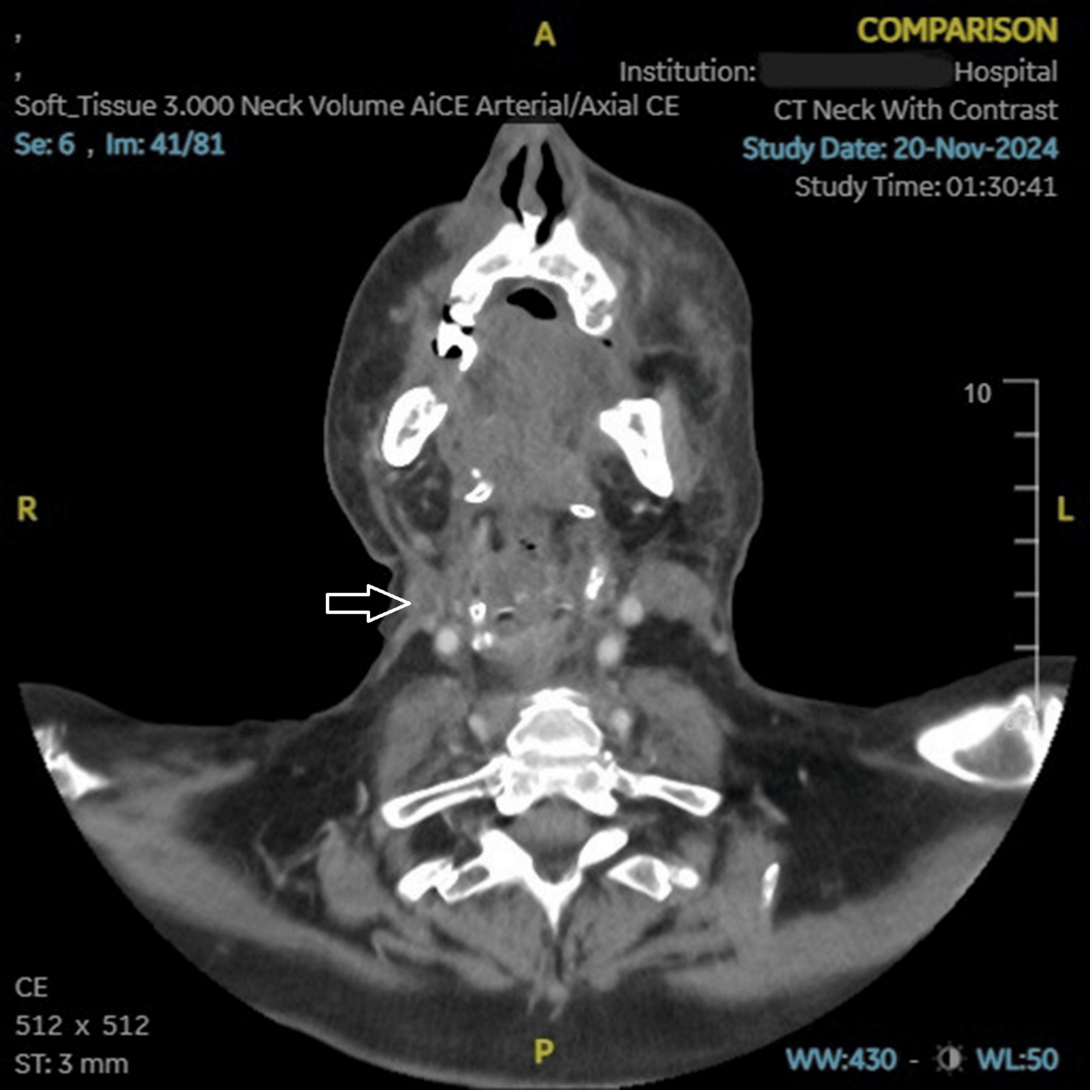
Contrast-enhanced arterial-phase CT of the neck demonstrating a non-opacified right IJV (arrow), consistent with right IJVT. The arrow points to the non-opacified right IJVT. IJVT: Internal jugular vein thrombosis, IJV: Internal jugular vein

The patient was initially treated for suspected facial cellulitis based on the clinical findings, with no response to treatment. The other differential was progression of malignancy, for which a short course of empiric intravenous dexamethasone was administered. To assess for disease progression, we repeated imaging. A contrast-enhanced arterial-phase CT of the neck and thorax showed a non-enhancing, non-expansile right IJV filling defect without continuity with tumour or nodal deposit, favouring bland thrombus (Figure 2). There was no evidence of another primary tumour elsewhere in the body or any metastases from the existing tumour, no abscess in the deep spaces of the neck was appreciated, no significant neck lymphadenopathy and no focal destructive bone lesion (ruling out clavicular osteomyelitis given the grade III ulcer over the right clavicular region) were identified. The CT also showed patchy atelectasis in both lower lung lobes; no consolidation or ground-glass opacification was appreciated, ruling out pneumonia.

The patient had no history of previous thromboembolic episodes, recent H&N surgery, deep neck space infections, recent local trauma or recent central venous catheterisation. There was no haematological condition, intravenous drug use, or renal, hepatic, or gastrointestinal pathology in the background, nor any risk factor other than the known H&N malignancy, making him prone to a hypercoagulable state. Family history was not remarkable for blood dyscrasias, thrombotic or metabolic risks.

Anticoagulation was initiated for a probable provoked IJVT, starting with weight-adjusted therapeutic enoxaparin (1.5 mg/kg once daily, subcutaneously), followed by apixaban (5 mg twice daily, oral). A CT pulmonary angiograph was performed to rule out pulmonary embolism, which was normal. After the initial management with anticoagulation, the patient showed significant symptomatic improvement. Unfortunately, later on, the patient passed away due to recurrent aspiration pneumonia because of non-compliance with advised tracheostomy care.

## Discussion

Clinical anatomy

The tongue is a conical structure comprised of extrinsic and intrinsic muscles, covered in papillae which give the tongue its characteristic velvet appearance [3]. It is divided into three parts, namely the tip, body, and base. The base of the tongue, also known as the pharyngeal part, is covered by lymphatic follicles forming the lingual tonsil [3,4]. The vascular supply to the tongue is through the lingual artery and vein, which drains into the common facial vein or IJV. The lymphatic drainage is through apical, marginal, central, and basal vessels, which drain into submental nodes later to the submandibular nodes and to the deep cervical nodes [3,4]. Basaloid SCC is a rare and aggressive high-grade variant of SCC found in the oropharynx [5].

Venous thromboembolism

Venous thromboembolism (VTE), blood clot formation within the venous system, is a common multifactorial condition. Virchow’s triad characterises the factors that contribute to thrombus formation: hypercoagulability, stasis or disturbed blood flow, and endothelial dysfunction. Malignancy engages Virchow’s triad, predisposing individuals to an increased risk of VTE [6,7].

Trousseau first described the connection between malignancy and thromboembolic events in 1865 [8], resulting in his eponymous syndrome, noting that unexpected or migratory thrombophlebitis can precede visceral malignancy. This has since been expanded to include chronic disseminated intravascular coagulopathy associated with microangiopathy or small vessel disease and arterial emboli [7,8]. The term has been adapted to include patients with advanced malignancy who develop thrombosis [6,9], as described in this case report.

Internal jugular vein thrombosis is a clinically significant complication in oncologic patients. Particularly of note in those with local malignancy, such as (H&N) cancers, they can present with neck swelling, erythema, neck pain, facial swelling, and fever [2,9]. There have been multiple reported cases of IJVT associated with H&N cancers [7,8] that are predominantly SCC [2,9,10].

VTE and H&N cancers

Cervical site VTE is rare and can lead to life-threatening complications such as pulmonary embolism, cerebral events, and superior vena cava obstruction, which should all be actively excluded in known cases of IJVT [6]. The risk of VTE varies by tumour site, stage, and histological grade [3]. Head and neck cancers, particularly SCC, are associated with high thrombosis risk and exhibit prothrombotic mechanisms such as tumour tissue factor, procoagulant protein expression, altered thrombosis and fibrinolysis, procoagulant cytokine production, and liberation of procoagulant microparticles [9]. However, the incidence of VTE in H&N cancer is low, between 0.16% and 3.125%, as demonstrated by Haen et al. Hence, the bland IJVT in our case is best understood as cancer-associated thrombosis (CAT) in the background of H&N SCC, driven by systemic prothrombotic state as well as local venous factors, in the absence of other risk factors [2,9]. A thrombophilia screen was not performed in our case, as thrombosis was likely provoked by malignancy in the background, based on the National Institute for Health and Care Excellence (NICE) NG158 VTE guideline [11].

Clinical significance

Internal jugular vein thrombosis is a dangerous and often neglected possible cause of common symptoms such as fever, facial swelling, and pain in an H&N cancer patient [2]. It may masquerade as cellulitis (fever, swelling, and facial pain) in H&N SCC. Due to the low incidence of VTE in H&N cancer [2,9], a physician may not initially investigate for a thrombus but may consider relevant tests in the presence of risk factors, such as malignancy [9]. Comparable cases describe IJVT initially attributed to infective presentations in H&N cancer, underscoring the value of early imaging when symptoms persist [11,12]. Minimal resources are required to perform a low-burden test, such as an ultrasound of the neck, which also serves as the first-line test for this condition [2,9]. To avoid complications, this justifies investigating IJVT as an important differential in such cases.

As per current guidelines, prophylactic anticoagulation is not routinely indicated for cancer patients, including those with local H&N malignancy, unless additional risk factors are present [13]. However, our case highlights that even in the absence of systemic risk factors other than malignancy itself, systemic procoagulant inclination and local tumour effects may provoke internal jugular vein thrombosis. This reinforces the need to maintain a high index of suspicion and to consider early imaging in such cases rather than relying solely on risk stratification. Scerrati underscores that IJVT is under-recognised, recommends ultrasound as the first-line test, with cross-sectional venography when needed, and highlights the importance of timely treatment to avoid complications [2]. In our patient, IJVT was suspected on an arterial-phase CT. Although dedicated venous-phase imaging or Doppler ultrasound is required to confirm diagnosis, arterial-phase CT, though suboptimal, demonstrated a non-enhancing, non-expansile, and clear intraluminal filling defect with complete lack of contrast opacification in our case. This finding, together with compatible symptoms, justified the need to commence anticoagulation. The initial cellulitis treatment delayed the diagnosis; hence, our threshold for treatment was lower once the intraluminal defect was found. Venous-phase CT and ultrasound were not performed for initial confirmation. The decision prioritised timely treatment to mitigate the risk of complications, in line with the emphasis on early imaging and management of IJVT [2].

Case literature similarly advocates prompt imaging and anticoagulation once IJVT is suspected in H&N cancer, even when first detected on non-targeted scans [10]. Neck Doppler ultrasound is the recommended first-line diagnostic modality for internal jugular vein thrombosis, with CT or MR venography reserved for confirmatory or problem-solving purposes when ultrasound is inconclusive or limited [2]. In this case, a confirmatory Doppler ultrasound or venous-phase CT was not performed due to clinical urgency following delayed recognition, which represents a limitation and may reduce diagnostic certainty. Although HPV/p16 status is routinely assessed in oropharyngeal SCC because of its established prognostic significance, it was not performed in this case, as the results would not have influenced management. Given the patient’s declining performance status and palliative treatment intent, radiotherapy was pursued for symptom control, and biomarker profiling was therefore not deemed clinically contributory [14,15].

Furthermore, it is also imperative to minimise iatrogenic risk at all stages of patient management in such cases, e.g., central venous catheterisation. Although our patient did not require central catheterisation, it is important to minimise iatrogenic risk when central venous access is necessary in such patients. Careful site selection (with a preference for the right internal jugular vein) and optimal catheter tip placement (near the superior vena cava-right atrial junction) may help reduce trauma and stasis, as well as the risk of catheter-related thrombosis. However, decisions should be individualized based on patient anatomy and clinical context [2,16].

## Conclusions

To the best of our knowledge, this case highlights an under-recognised presentation of provoked IJVT in H&N SCC. This stresses the value of prompt imaging and timely anticoagulation. Clinicians should consider cancer-associated thrombosis in the differential diagnoses when patients present with unexplained cervical or facial swelling. Early detection and treatment of the condition can be crucial in preventing life-threatening complications.
